# Assessment of target-mediated uptake with immuno-PET: analysis of a phase I clinical trial with an anti-CD44 antibody

**DOI:** 10.1186/s13550-018-0358-8

**Published:** 2018-01-22

**Authors:** Yvonne W. S. Jauw, Marc C. Huisman, Tapan K. Nayak, Danielle J. Vugts, Randolph Christen, Valerie Meresse Naegelen, Dominik Ruettinger, Florian Heil, Adriaan A. Lammertsma, Henk M. W. Verheul, Otto S. Hoekstra, Guus A. M. S. van Dongen, C. Willemien Menke-van der Houven van Oordt

**Affiliations:** 1 Department of Hematology, VU University Medical Center, Amsterdam, the Netherlands; 2 Department of Radiology and Nuclear Medicine, VU University Medical Center, Amsterdam, the Netherlands; 3 Department of Pharma Research and Early Development, Roche Innovation Center, Basel, Switzerland; 4 Department of Product Development, Safety Risk Management, Roche, Basel, Switzerland; 5 Department of Pharma Research and Early Development, Roche Innovation Center, Munich, Germany; 6 Department of Medical Oncology, VU University Medical Center, Amsterdam, the Netherlands

**Keywords:** PET, Antibody, Anti-CD44 humanized antibody, RG7356, Molecular imaging

## Abstract

**Background:**

Ideally, monoclonal antibodies provide selective treatment by targeting the tumour, without affecting normal tissues. Therefore, antibody imaging is of interest, preferably in early stages of drug development. However, the imaging signal consists of specific, as well as non-specific, uptake. The aim of this study was to assess specific, target-mediated uptake in normal tissues, with immuno-PET in a phase I dose escalation study, using the anti-CD44 antibody RG7356 as example.

**Results:**

Data from thirteen patients with CD44-expressing solid tumours included in an imaging sub-study of a phase I dose escalation clinical trial using the anti-CD44 antibody RG7356 was analysed. ^89^Zirconium-labelled RG7356 (1 mg; 37 MBq) was administered after a variable dose of unlabelled RG7356 (0 to 675 mg). Tracer uptake in normal tissues (liver, spleen, kidney, lung, bone marrow, brain and blood pool) was used to calculate the area under the time antibody concentration curve (AUC) and expressed as tissue-to-blood AUC ratios.

Within the dose range of 1 to 450 mg, tissue-to-blood AUC ratios decreased from 10.6 to 0.75 ± 0.16 for the spleen, 7.5 to 0.86 ± 0.18 for the liver, 3.6 to 0.48 ± 0.13 for the bone marrow, 0.69 to 0.26 ± 0.1 for the lung and 1.29 to 0.56 ± 0.14 for the kidney, indicating dose-dependent uptake. In all patients receiving ≥ 450 mg (*n* = 7), tumour uptake of the antibody was observed.

**Conclusions:**

This study demonstrates how immuno-PET in a dose escalation study provides a non-invasive technique to quantify dose-dependent uptake in normal tissues, indicating specific, target-mediated uptake.

**Electronic supplementary material:**

The online version of this article (10.1186/s13550-018-0358-8) contains supplementary material, which is available to authorized users.

## Background

Treatment of cancer has improved as a result of immunotherapy with monoclonal antibodies (mAbs). Ideally, mAbs selectively target tumour cells, resulting in limited toxicity compared to classical chemotherapy.

However, lack of mAb selectivity may result in significant toxicity and/or suboptimal tumour targeting, leading to therapy failure. Therefore, it is important to confirm tumour selectivity of a novel candidate mAb to minimize toxicity and maximize efficacy, preferably in early stages of drug development. Currently, toxicity is assessed by dose escalation in traditional phase I trials, using dose-limiting toxicity and a maximum tolerated dose to establish the therapeutic dose for the next stages of drug development (phase II and III trials). For the ideal mAb, selective tumour targeting is expected, with limited target antigen-mediated specific uptake in normal tissues.

**Fig. 1:**
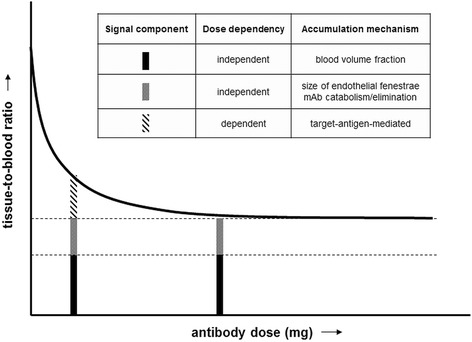
Immuno-PET signal components in a phase I dose escalation study. The tissue-to-blood ratio is shown as a function of administered antibody dose. For example, target antigen-mediated uptake is dose-dependent, while blood volume fraction, catabolism or elimination are dose-independent accumulation mechanisms

Recently, there is increasing interest in the use of imaging techniques to measure the mAb biodistribution in vivo without requiring blood or tissue samples [1]. After inert and stable radiolabelling, the radioactive mAb can be used to study the biodistribution of the non-radioactive mAb. According to this principle, positron emission tomography (PET) with ^89^Zr-labelled mAbs provides a non-invasive tool for in vivo visualization and quantification of mAbs [2–4]. Quantification of antibody accumulation in normal tissues and tumour using PET imaging can be an important non-invasive tool to evaluate the therapeutic potential of antibodies and antibody conjugates. For this purpose, target-mediated specific uptake is of interest. However, the measured PET signal comprises non-specific uptake (dependent on the tissue blood volume fraction, as well as other tissue characteristics, for example, size of endothelial fenestrae by which the antibody passes through the capillary wall) and potentially target antigen-mediated specific uptake. Differentiation between these specific and non-specific contributions to the PET signal is possible, if we assume that they are dose-dependent and dose-independent, respectively (Fig. 1). In this paper, we present an experimental approach to assess specific uptake with immuno-PET in a dose escalation study, using RG7356 as an example.

Investigational RG7356 is an anti-CD44 recombinant humanized mAb, which targets the constant region of the extracellular domain of CD44 and provides antibody-dependent cellular phagocytosis of the malignant cells by macrophages [5]. CD44 is a human cell-surface glycoprotein, which is expressed by several solid tumours as well as cancer stem cells and has a role in cell proliferation, migration and angiogenesis. This target antigen has been considered attractive for immunotherapy [6], as blocking inhibits tumour growth and metastatic potential [7, 8]. A preclinical dose escalation study with ^89^Zr-labelled RG7356 confirmed tumour targeting of CD44+ tumours in xenograft-bearing mice. Since RG7356 is not cross-reactive with murine CD44, studies in mice do not provide any information regarding accessible binding sites in physiologically normal organs. Assessment of biodistribution in cynomolgus monkeys showed uptake in normal CD44+ tissues, for example, spleen and bone marrow [9]. Subsequently, a first-in-human phase I dose escalation trial was performed in patients with advanced, CD44-expressing solid tumours, showing that RG7356 was well tolerated with modest clinical efficacy [10]. PET imaging with ^89^Zr-labelled RG7356 was performed to assess biodistribution and tumour uptake of RG7356 in a subgroup (13 of 65 patients) of this phase I trial. The previous publication on the main trial [10] included the background of the antigen and antibody as well as a brief summary of visual assessment of the imaging subgroup. ^89^Zr-RG7356 was localized to the spleen, bone marrow and liver, while tumour accumulation of ^89^Zr-RG7356 was observed with co-administration of unlabelled antibody (≥ 199 mg), suggesting that the modest efficacy was not related to poor drug delivery to the tumour per se [10]. In the current study, we separately report on the exploratory imaging sub-study with ^89^Zr-labelled RG7356 by performing quantitative analysis of the imaging data.

The aim of this study was to quantify biodistribution and tumour uptake of ^89^Zr-RG7356 and to evaluate whether uptake of ^89^Zr-RG7356 in normal tissues was dose-dependent as indication of target-mediated specific uptake. With this example, we aim to provide a general approach for application of immuno-PET to evaluate new drug-target-combinations in first-in-human studies.

## Methods

### Patient population

Patients with advanced CD44-expressing solid tumours were included in a multicentre phase I clinical study with RG7356 (ClinicalTrials.gov Identifier NCT01358903) [10]. As part of the screening procedure, tumour biopsies were obtained and analysed centrally by Ventana Medical Systems Inc. (VSMI, Tucson, AZ, USA), using clone SP37 as anti-CD44 primary antibody to assess tissue quality, tumour content and CD44 expression by immunohistochemistry (IHC). Patients with a IHC score of ≥ 1+ for CD44 positivity on the pretreatment biopsy were included. For specific scoring criteria for CD44 expression in patient samples, see Additional file 1: Table S1. Study design and inclusion and exclusion criteria have been reported previously [5]. This study was approved by the Medical Ethics Review Committee of the VU University Medical Center, Amsterdam, and performed in accordance with the Declaration of Helsinki. All study-related procedures were performed after patients gave their written informed consent. Patients enrolled in this phase I study at the VU University Medical Center were asked to participate in the exploratory imaging sub-study.

### PET imaging study design

For PET imaging, RG7356 (Roche, Basel, Switzerland) was labelled with ^89^Zr (BV Cyclotron VU, Amsterdam, the Netherlands) according to Good Manufacturing Practice (GMP) standards, as previously described [11–13]. Information on the quality control of the radiolabelled tracer can be found in the Additional file 2: Supplementary Data. Patients received ~ 37 MBq ^89^Zr-labelled RG7356 (1 mg) within 2 h after administration of a variable dose (range 0–675 mg) of unlabelled RG7356. Pre-loading with unlabelled mAb was preferred from a logistical perspective (infusion of the unlabelled mAb in a phase I oncology unit), assuming no difference in uptake between pre-loading and co-infusion.

Whole body PET and low dose CT (ldCT) scans were acquired on a Gemini TF-64 PET/CT scanner (Philips Healthcare, Best, the Netherlands) and scheduled at 1, 24 and 96 h post injection (p.i.). Images were reconstructed as described previously [14]. After completing the imaging procedure, patients continued in the main study and received RG7356 in the highest dose cohort that was cleared for safety.

### ^89^Zr-RG7356 PET analysis

Visual assessment of biodistribution and tumour uptake was performed by a nuclear medicine physician. Tumour uptake of ^89^Zr-RG7356 was defined as focal uptake exceeding local background. PET scans were considered positive if at least one tumour lesion showed ^89^Zr uptake at 96 h p.i..

Volumes of interest (VOIs) of the liver, spleen, kidney, lung, bone marrow, blood pool and brain were delineated to derive mean activity concentrations (AC_mean_ in Bq mL^− 1^). For the lung and brain, VOIs were semi-automatically defined on the ldCT and projected on the PET images. The thresholds for lung VOIs were based on Hounsfield units (lower limit − 1000, upper limit − 400), therefore excluding tissue with higher Hounsfield units (e.g. tumour localisations in the lung). VOIs of the liver, spleen and kidney were manually delineated on the PET images themselves, using the ldCT as reference. Fixed sized VOIs with volumes of 8.6 and 2.9 mL were placed on lumbar vertebrae and aortic arch (on ldCT) to estimate AC_mean_ in the bone marrow and blood pool, respectively. Tumour lesions were manually delineated, and peak activity concentrations (AC_peak_) were derived per tumour VOI [15]. Assuming that tumour uptake is only due to non-specific uptake (blood volume fraction), the tumour blood volume fraction can be estimated by dividing the AC in the tumour by the AC in the blood pool.

For all VOIs, standardized uptake values (SUVs) were calculated by dividing AC (decay corrected to time of injection) by injected dose (ID in Bq) corrected for body weight. Additionally, all radioactivity concentrations were converted to antibody concentrations by the following formula: *C*_mAb_ = (AC/ID) × *D*_mAb_, where *C*_mAb_ is the mAb concentration in mg/mL and *D*_mAb_ is the total (= labelled + unlabelled) antibody dose administered in milligrams.

All measured time points were combined to derive a time antibody concentration curve. Subsequently, using trapezoidal integration, the AUC in mg h mL^− 1^ was calculated. For each tissue, the tissue-to-blood AUC ratio was obtained by dividing the tissue AUC by the blood pool AUC. Data, averaged over the number of patients per dose cohort, are presented as mean ± SD.

## Results

### Patient characteristics

Thirteen patients with advanced CD44-expressing solid tumours were included. Five cohorts with different doses of antibody were evaluated: 1 mg (*n* = 1), 100 mg (*n* = 3), 200 mg (*n* = 2), 450 mg (*n* = 5) and 675 mg (*n* = 2). These dose cohorts were determined based on safety as assessed in the main phase I trial. Subsequently, the imaging dose was increased when imaging and tumour characteristics were not satisfactory (e.g. no visible tumour uptake due to uptake in normal tissues/high background). Patient details are provided in Table 1. In each case, CD44 expression was confirmed by central reviewing of tumour biopsies (Additional file 3: Figure S1).

**Table 1:** Patient details

Patient	Gender	Tumour type	CD44 expression (IHC)		
			Membrane score	H-score	Biopsy location
1	F	Squamous cell carcinoma cervix	3	285	Lung
2	M	Adenocarcinoma oesophagus	2 (focal)	30	Oesophagus
3	M	Colorectal carcinoma	2 (focal)	25	Liver
4	M	Melanoma	3	250	Liver
5	M	Colorectal carcinoma	2	130	Peritoneal
6	F	Squamous cell carcinoma cervix	2 (focal)	65	Cervix
7	M	Basaloid carcinoma tonsil	3	135	Lung
8	M	Squamous cell carcinoma oesophagus	3	210	Neck left
9	M	Colorectal carcinoma	2	85	Liver
10	M	Cholangiocarcinoma	3	200	Liver
11	F	Squamous cell carcinoma ear canal	3	260	Neck left
12	M	Colorectal carcinoma	2 (focal)	35	Liver
13	M	Colorectal carcinoma	3	218	Liver

### ^89^Zr-RG7356 PET: biodistribution

**Fig. 2:**
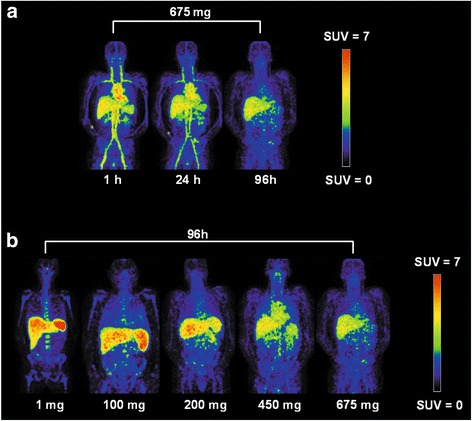
Biodistribution of ^89^Zr-labelled-RG7356. **a** As function of time: maximum intensity projections at 1, 24 and 96 h p.i. for patient 12 (675 mg dose cohort). **b** As function of total administered antibody dose: maximum intensity projections at 96 h p.i. for various dose cohorts (1, 100, 200, 450 and 675 mg)

Overall, visual assessment of PET images showed mainly blood pool activity of ^89^Zr-labelled-RG7356 at 1 h p.i., decreasing over time (Fig. 2a). Differences in biodistribution of ^89^Zr-labelled-RG7356 were observed for patients in the different dose cohorts. Intense tracer uptake in the spleen, liver and bone marrow was visualized for the lowest dose cohorts (1 and 100 mg), decreasing with increasing antibody doses (Fig. 2b).

**Fig. 3:**
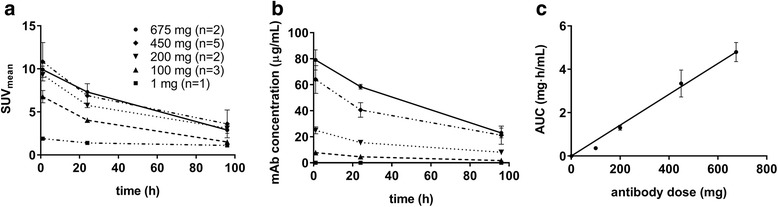
Blood pool concentrations of ^89^Zr-RG7356 and RG7356. **a** Radioactivity concentrations of ^89^Zr-RG7356 in blood pool as function of time. **b** Antibody concentrations of RG7356 in blood pool as function of time. **c** AUC of the time antibody concentration curve of RG7356 in blood pool as function of antibody dose. Data is presented as mean; error bars represent SD

The blood pool activity concentration of ^89^Zr-labelled-RG7356, expressed as SUV over time, is shown in Fig. 3a. This graph displays only the measured activity of the radioactive tracer. In Fig. 3b, derived absolute antibody concentrations in blood pool, which represent the total amount of antibody (labelled + unlabelled dose) are shown. The AUC of this time antibody concentration curve is shown as a function of the administered antibody dose in Fig. 3c. Dose-independent uptake would result in a linear relationship of AUC with antibody dose. Figure 3c does, overall, show this linear relationship. The 100 mg datapoint, however, has a measured AUC lower than expected based on this linear trend.

**Fig. 4:**
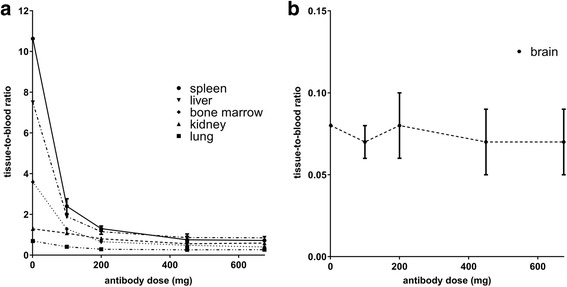
Tissue-to-blood AUC ratio of RG7356 as a function of antibody dose. **a** Ratios for spleen, liver, bonemarrow, kidney and lung. **b** Ratio for brain. Data is presented as mean; error bars represent SD

Tissue-to-blood AUC ratios are presented as a function of administered antibody dose in Fig. 4. For the brain, the tissue-to-blood AUC ratio was constant (0.07 ± 0.01) and independent of administered antibody dose. All other tissue-to-blood AUC ratios (spleen, liver, bone marrow, kidney and lung) were dependent of the antibody dose administered.

For the lowest dose cohorts (1 and 100 mg), relative high tissue-to-blood AUC ratios were observed. The spleen showed the highest tissue-to-blood AUC ratio for the 1 mg dose cohort (10.6), followed by the liver (7.5), bone marrow (3.6), kidney (1.3), lung (0.7) and brain (0.08). For liver, spleen and kidney, a constant tissue-to blood AUC ratio was reached for doses ≥ 450 mg and for the lung at doses ≥ 200 mg. For the 675 mg dose cohort, the liver showed the highest tissue-to-blood AUC ratio of 0.85 ± 0.08, followed by the spleen (0.72 ± 0.09), kidney (0.59 ± 0.08), bone marrow (0.40 ± 0.04), lung (0.27 ± 0.03) and brain (0.07 ± 0.02).

### ^89^Zr-RG7356 PET: tumour uptake

**Fig. 5:**
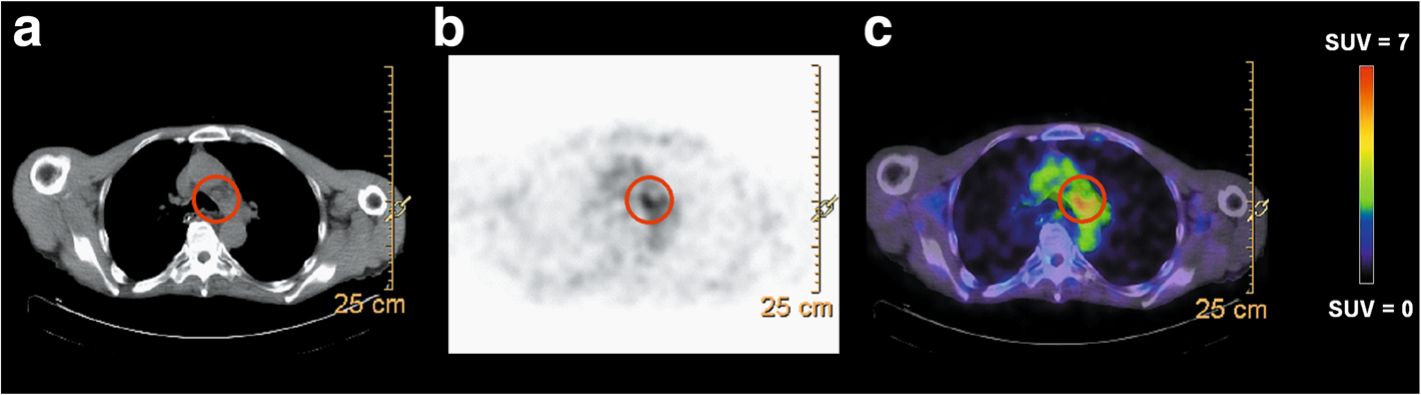
Example of tumour uptake of ^89^Zr-RG7356 at 96 h p.i.. Tumour lesion mediastinal/in the aorta–pulmonary window (patient 8, 450 mg cohort). **a** Low dose CT. **b** Attenuation-corrected PET. **c** Fused image

Assessment of tumour uptake of ^89^Zr-RG7356 at 96 h p.i. per tumour lesion is shown in Table 2. In all patients receiving ≥ 450 mg RG7356 (*n* = 7), tumour uptake was observed. An example image of visible tumour uptake is shown in Fig. 5. For this patient, the time activity curves for blood pool, normal tissues and tumour are provided as an example (Additional file 4: Figure S2). In 1 out of 6 patients receiving ≤ 200 mg RG7356, diffuse uptake of ^89^Zr-labelled-RG7356 was observed in the lung, indicating diffuse tumour localisation. Quantification was not feasible due to the diffuse localisation. Quantification of mAb uptake in focal tumour lesions resulted in an average SUV_peak_ of 3.7 ± 1.7 for the 450 mg cohort and 6.5 ± 1.3 for the 675 mg cohort. Average tumour AUC was 1.5 ± 0.5 mg h/mL for the 450 mg cohort and 3.2 ± 0.5 mg h/mL for the 675 mg cohort. Average tumour to blood AUC ratio was 0.46 ± 0.15 for the 450 mg cohort and 0.65 ± 0.07 for the 675 mg cohort. For the 675 mg cohort, the average tumour blood volume fraction was 67%.

**Table 2:** Tumour uptake of ^89^Zr-RG7356 and RG7356 at 96 h p.i.

Patient	Dose cohort (mg)	Visual assessment	Localisation	SUV_peak_	AUC (mg h/mL)	Tumour-to-blood AUC ratio
1	1	−	−	−	−	−
2	100	−	−	−	−	−
3	100	*	−	−	−	−
4	100	−	−	−	−	−
5	200	−	−	−	−	−
6	200	−	−	−	−	−
7	450	+	Brain L	1.8	0.35	0.09
			Lung R	8.4	2.06	0.55
8	450	+	Skull L	3.3	1.41	0.43
			Upper neck L	3.5	1.62	0.49
			Lower neck L	2.8	1.43	0.43
			Supraclavicular R	3.1	1.67	0.50
			Mediastinal	4.8	2.37	0.72
			Lung L	2.2	1.29	0.39
			Lung R1	2.1	1.06	0.32
			Lung R2	4.3	1.75	0.53
9	450	+	Lung L	1.8	0.89	0.32
			Lung R	2.2	0.99	0.36
			Rectum	5.6	1.75	0.64
10	450	+	Lung L	4.6	1.47	0.56
			Lung R	3.9	1.41	0.53
11	450	+	Mastoid L	4.5	1.81	0.42
12	675	+	Sigmoid L	4.9	2.55	0.59
13	675	+	Pelvic R	6.5	3.83	0.73
			Sacrum	8.1	3.29	0.63

*−* no visible tumour uptake

*Diffuse uptake in the lung

## Discussion

In this study, we assessed dose-dependent and dose-independent uptake of the ^89^Zr-labelled anti-CD44 antibody RG7356 in normal tissues to identify specific, target-mediated uptake on immuno-PET in a dose escalation phase 1 study.

Both dose-dependent and dose-independent uptake were observed, reflecting specific as well as non-specific uptake of RG7356. For tissues without antigen expression, a linear increase in antibody concentrations can be expected for increasing antibody doses, driven by perfusion and blood volume of the tissue. However, our results suggest a mechanism that extracts antibody from the blood pool to tissues, in addition to the non-specific uptake mechanisms (Fig. 3c). Therefore, tissue-to-blood AUC ratios were used to evaluate dose-dependent uptake of RG7356 for the following tissues: liver, spleen, bone marrow, kidney, lung and brain. For the brain, a constant low tissue-to-blood AUC ratio was observed for all dose cohorts. Assuming that RG7356 does not cross the blood–brain barrier, this value is determined by the blood volume fraction of the brain. For the spleen, liver, bone marrow, kidney and lung, dose-dependent uptake of ^89^Zr-RG7356 was observed, indicating target antigen-mediated specific uptake in these tissues. A very similar pattern of dose-dependent uptake in the spleen, liver and bone marrow has been reported previously in the preclinical study with ^89^Zr-RG7356 in cynomolgus monkeys, indicating that such preclinical immuno-PET studies can be predictive with respect to normal tissue uptake in human [9].

Target antigen expression in these tissues is a plausible explanation for dose-dependent uptake, as protein expression of CD44 has been reported for normal bone marrow, spleen, lung, kidney and liver (bile ducts) [16, 17]. Although dose-dependent uptake in tissues was observed, a constant tissue-to-blood AUC ratio was reached at 450 mg for all tissues, indicating target antigen saturation.

In addition, dose-independent uptake of the tracer in the liver, spleen, bone marrow, kidney and lung was observed, indicating non-specific uptake. For the liver, based on a 30% blood volume fraction [18], a liver-to-blood AUC ratio of 0.3 would be expected. However, we observed a liver-to-blood AUC ratio of 0.85 ± 0.08 for the 675 mg dose cohort. The difference between the tissue-to-blood AUC ratio and blood volume fraction represents an additional accumulation mechanism in the liver, for example, the large endothelial fenestrae or antibody catabolism. Stability of ^89^Zr-labelled antibodies, with minimal release of ^89^Zr, has been demonstrated in many in vitro and in vivo preclinical as well as clinical studies [11, 19, 20]. There are no experimental data supporting accumulation of free ^89^Zr in normal tissues, except for the observation that free ^89^Zr, arising after internalisation and intracellular catabolism of the conjugate, may accumulate in bone tissue (not bone marrow) [19]. However, in our study, we did not observe ^89^Zr accumulation in the bone (Fig. 2).

Although dose-dependent, as well as dose-independent, uptake in normal tissues was found in this imaging study, there were no safety concerns in the corresponding phase I dose escalation study, with treatment doses up to 1500 mg biweekly/2250 mg weekly. The overall safety profile of RG7356 was acceptable. Dose-limiting toxicities included febrile neutropenia and aseptic meningitis [10]. However, this phase I study was terminated at an early stage due to the lack of evidence of a clinical and/or pharmacodynamic (PD) dose–response relationship with RG7356.

We observed tumour uptake in all patients receiving ≥ 450 mg, with an extremely high tumour blood volume fraction estimated for the 675 mg cohort. Although this might suggest target-mediated specific tumour uptake, another study design would have been more informative on this point [21]. This requires measurement of the same tumour lesion after administration of different antibody doses to exclude differences in tumour characteristics, for example, blood volume fraction. Learning from the present study, we recently demonstrated target-mediated specific tumour uptake in a PET imaging study with an anti-HER3 mAb in which the ^89^Zr-labelled antibody was administered twice (with a variable dose of unlabelled antibody) to a single patient [21]. Although biopsies taken after the immuno-PET could have provided additional confirmation with immunohistochemistry, this was not included in the study design due to the fragile patient population.

No focal tumour uptake was visualized in the lowest dose cohorts (1–200 mg). This observation cannot be explained by the level of CD44 expression or the percentage of CD44-positive tumour cells (Table 1). A probable explanation why tumour visualization is hampered for the lowest dose cohorts is that dose-dependent uptake in normal tissues leads to lower visual tumour contrast, assuming similar binding constants and accessibilities of the target antigen in both normal tissues and tumour. However, in the higher dose cohorts, differences between binding constant became apparent where the dose-dependent tracer uptake in normal tissues does not significantly contribute to the imaging signal anymore (Fig. 1); target antigen-mediated tracer uptake will result in sufficient visual contrast to allow identification and quantification of tumour targeting.

In this study, PET imaging with the novel anti-CD44 monoclonal antibody RG7356 confirmed tumour uptake for patients receiving ≥ 450 mg. However, dose-dependent uptake of RG7356 in normal tissues indicates target antigen expression, limiting the use of RG7356 for targeting toxic payloads to the tumour like in antibody–drug conjugate (ADC) approaches.

This exploratory imaging study demonstrates how immuno-PET with a ^89^Zr-labelled mAb can be used as a general method during phase I dose escalation studies to evaluate the therapeutic potential of an antibody. Evaluation of dose-dependent and dose-independent normal tissue uptake with immuno-PET reflects specific and non-specific uptake. Antibody quantification obtained by molecular imaging provides an additional, non-invasive method to study in vivo mAb biodistribution, besides traditional PK obtained by blood sampling. Especially for a candidate mAb with a potential future as ADC, the resulting information (prevention of potential toxicity/additional development costs) may justify the patient burden and cost for additional scans in a limited number of patients in a phase I setting.

For a mAb which continues in further stages of drug development, in vivo measurements of antibody concentrations in tissue and tumour can be of value for PK/PD modelling/dose optimization and response prediction to guide individualized treatment.

## Conclusions

This study demonstrates how immuno-PET in early clinical drug development provides a non-invasive technique to quantify dose-dependent uptake in normal tissues, indicative of specific, target-mediated uptake.

## Additional files

Additional file 1: Table S1. Specific scoring criteria for CD44 expression in patient samples. (DOCX 14 kb)

Additional file 2: Supplementary Data. Information on the quality control of the radiolabelled tracer. (DOCX 16 kb)

Additional file 3: Figure S1. Example of immunohistochemistry staining for CD44 for (a) patient 9, rectum carcinoma, biopsy of a liver metastasis CD44 score: 2+; (b) patient 8, squamous cell carcinoma of the head and neck, biopsy of a neck metastasis, CD44 score: 3+. (TIFF 4368 kb)

Additional file 4: Figure S2. Example graph of blood pool (a), normal tissue (b) and tumour (c) for patient 8. The mediastinal tumour (patient 8) corresponds with Fig. 5. The tumour in the skull L (patient 8) corresponds with Additional file 5: Figure S3. (TIFF 358 kb)

Additional file 5: Figure S3. Additional examples of focal tumour uptake of ^89^Zr-RG7356 at 96 h p.i.. From left to right: low dose CT, attenuation-corrected PET and fused image. (a) Tumour lesion: left side of the skull (patient 8, 450 mg cohort). (b) Tumour lesion: sacrum (patient 13, 675 mg cohort). (TIFF 6335 kb)
