# Supplementary material for: Assessment of target-mediated uptake with immuno-PET: analysis of a phase I clinical trial with an anti-CD44 antibody
Source: EJNMMI Res. 2018 Jan 22;8:6. doi: 10.1186/s13550-018-0358-8 (PMC5778091; doi:10.1186/s13550-018-0358-8)
Supplement: Supplementary file 1 — Specific scoring criteria for CD44 expression in patient samples. (DOCX 14 kb) [file 13550_2018_358_MOESM1_ESM.docx]

**Supplemental Table 1** Specific scoring criteria for CD44 expression in patient samples

| Staining Intensity in tumour cells | CD44 Scoring Assignment | Eligible for inclusion in trial |
| --- | --- | --- |
| 3+ in >30% of tumour cells | 3 | Yes |
| 2+ in >30% of tumour cells | 2 | Yes |
| 2+ or 3+ in <30% of tumour cells | 2 (focal) | Yes |
| (1+) or 1+ in >30% of tumour cells | 1 | Yes |
| (1+) or 1+ in <30% of tumour cells | 1 (focal) | No |
| No staining present in tumour cells | 0 | No |
| Indeterminate | Determination of reactivity not possible. Includes necrosis, no tumour present, no tissue present, or artifacts. | No |
