# Supplementary material for: Assessment of target-mediated uptake with immuno-PET: analysis of a phase I clinical trial with an anti-CD44 antibody
Source: EJNMMI Res. 2018 Jan 22;8:6. doi: 10.1186/s13550-018-0358-8 (PMC5778091; doi:10.1186/s13550-018-0358-8)
Supplement: Supplementary file 2 — Information on the quality control of the radiolabelled tracer. (DOCX 16 kb) [file 13550_2018_358_MOESM2_ESM.docx]

Supplementary Data – Information on the quality control of the radiolabelled tracer.

^89^Zr-RO5429083 has been produced in compliance with current Good Manufacturing Practice at the VU University Medical Center. The procedures of radiolabelling of RO5429084 with 89Zr have been validated with respect to quality controls and the final quality of the product. ^89^Zr-RO5429083 has been produced according to previously reported method (1).  In short, 1 mL RO5429083 (10 mg/mL) was rebuffered to 0.9% NaCl by size-exclusion chromatography (PD10, GE Healthcare). The pH of 5 mg/mL (1 mL) RO5429083 in 0.9% NaCl was adjusted to 9.5-9.7 with 0.1 M Na_2_CO_3_ after which 20 µL of 3.3 mM (2 equivalents) Fe-TFP-N-suc-DFO-ester in acetonitrile was added. The solution was left for 30 minutes at room temperature, followed by the addition of 50 µL of 100 mg/mL gentisic acid pH 4.0-4.2 and adjustment of the pH to 4.2-4.5 with 0.25 M H2SO4. Next 50 µL of 25 mg/mL EDTA was added and the reaction incubated for 30 minutes at 37˚C. Next, the conjugated DFO-RO5429083 was purified by size exclusion chromatography (PD10, GE Healthcare) and the product collected in 5 mg/mL gentisic acid pH = 5.1 ±0.2. Finally DFO-RO5429083 was radiolabelled. To this end 200 µL 1M oxalic acid containing the required amount of ^89^Zr was mixed with 90 µL 2M Na_2_CO_3_ and reacted for 3 minutes. Next 1 mL 0.5 M Hepes, 0.11 mL 0.9% NaCl and 0.6 mL DFO-RO5429083 (~1.5 mg) were added and reacted for 60 minutes at room temperature while slowly shaken. After the incubation period 89Zr-RO5429083 was purified by size exclusion chromatography using a PD10 column. The product was eluted in 5 mg/mL gentisic acid pH = 5.1 ±0.2. The product was formulated to arrive at an injection dose of 37 MBq – 1 mg – 20 mL ^89^Zr-RO5429083. The mean of the product pH was 5.04 ± 0.08. The mean radiochemical purity as assessed by iLTC was 97.7 ± 1.3%. To this end 2 µL of product was pipetted on a Biodex chromatography strip (150-771) and developed with 50 mM citric acid pH 5.0 containing 10% acetonitrile. The strip was cut at the indicated line and the bottom part contained ^89^Zr-RO5429083 and the top part contained free ^89^Zr and ^89^Zr-DFO.

The mean radiochemical purity was 97.9 ± 2.2 as determined by size exclusion HPLC using a superdex 200 10/30 GL size exclusion column (GE healthcare Life sciences) including a guard column using a mixture of 0.05 M sodium phosphate, 0.15 M sodium chloride (pH 6.8) and 0.01 M NaN_3_ as the eluent at a flow rate of 0.5 mL/min. The mean immune reactive fraction as assessed by Lindmo binding assay was 76.3 ± 3.1%. Sterility of each ^89^Zr-GSK2849330 batch was assured by performing a media fill immediately after final filter sterilisation of each batch. These procedures resulted in a sterile final product with endotoxin levels <0.3 EU/mL.

1. Verel I, Visser GW, Boellaard R, Stigter-van Walsum M, Snow GB and Van Dongen GA. 89Zr immuno-PET: comprehensive procedures for the production of 89Zr-labeled monoclonal antibodies. J. Nucl. Med. 2003;44, 1271-1281.
