# Supplementary figures and images for: Assessment of target-mediated uptake with immuno-PET: analysis of a phase I clinical trial with an anti-CD44 antibody
Source: EJNMMI Res. 2018 Jan 22;8:6. doi: 10.1186/s13550-018-0358-8 (PMC5778091; doi:10.1186/s13550-018-0358-8)

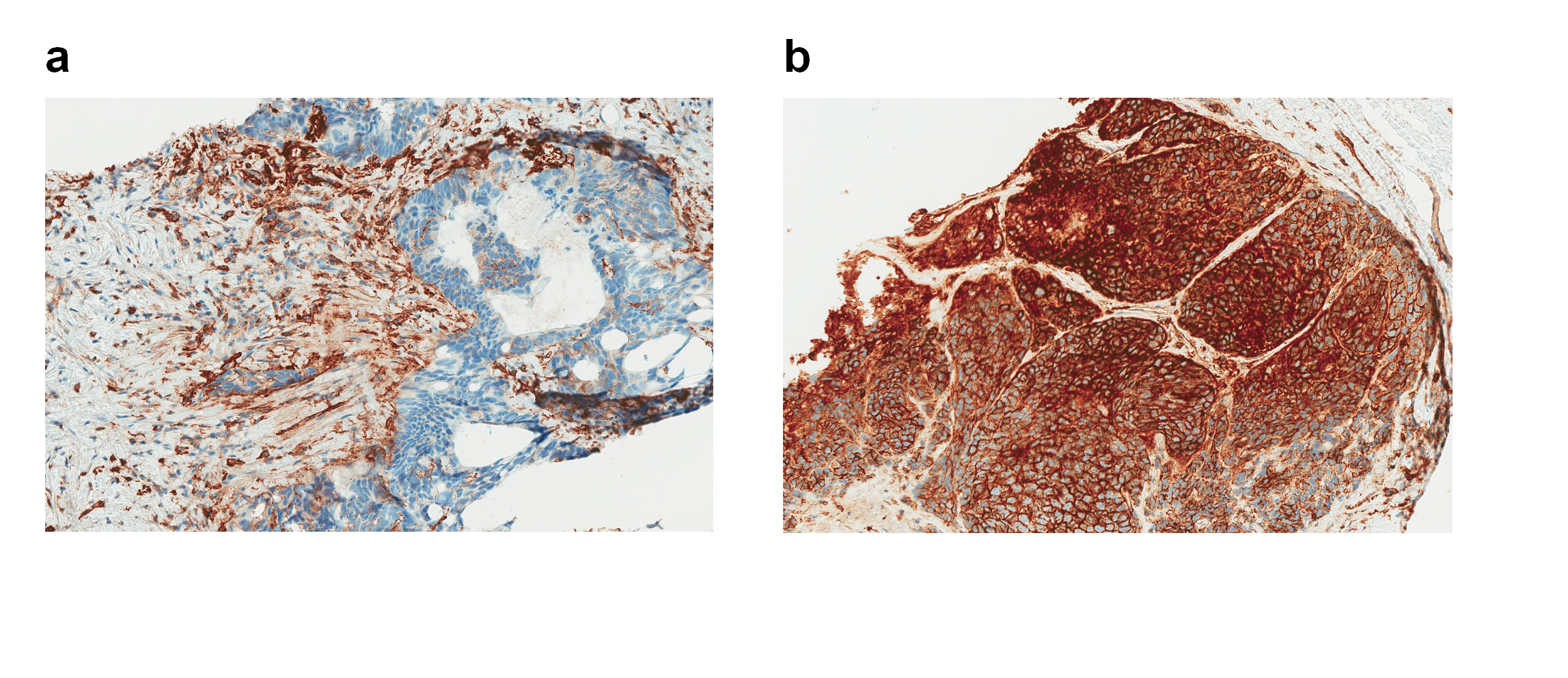

Supplement: Supplementary file 3 — Example of immunohistochemistry staining for CD44 for (a) patient 9, rectum carcinoma, biopsy of a liver metastasis CD44 score: 2+; (b) patient 8, squamous cell carcinoma of the head and neck, biopsy of a neck metastasis, CD44 score: 3+. (TIFF 4368 kb) [file 13550_2018_358_MOESM3_ESM.tif]

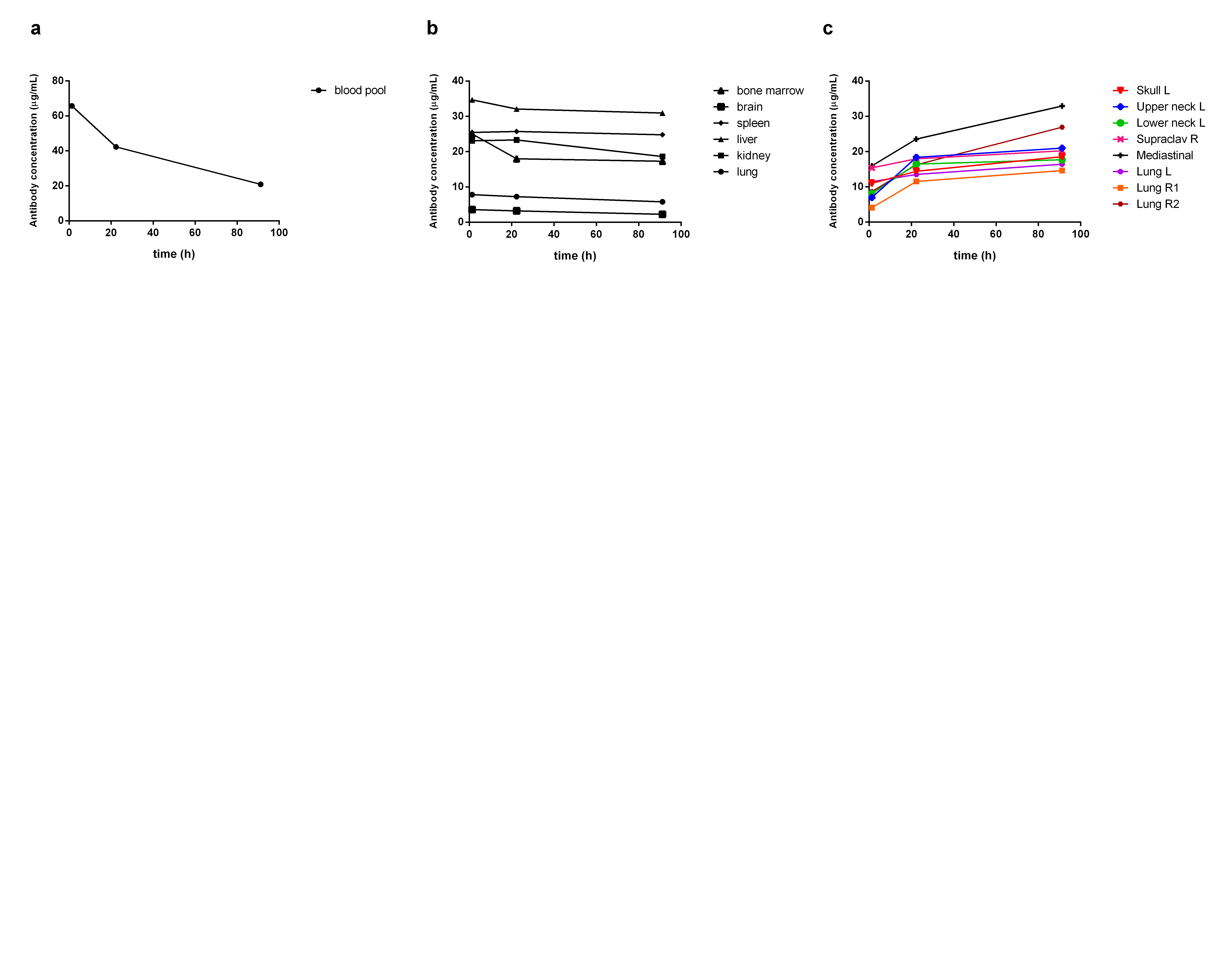

Supplement: Supplementary file 4 — Example graph of blood pool (a), normal tissue (b) and tumour (c) for patient 8. The mediastinal tumour (patient 8) corresponds with Fig. 5. The tumour in the skull L (patient 8) corresponds with Additional file 5: Figure S3. (TIFF 358 kb) [file 13550_2018_358_MOESM4_ESM.tif]

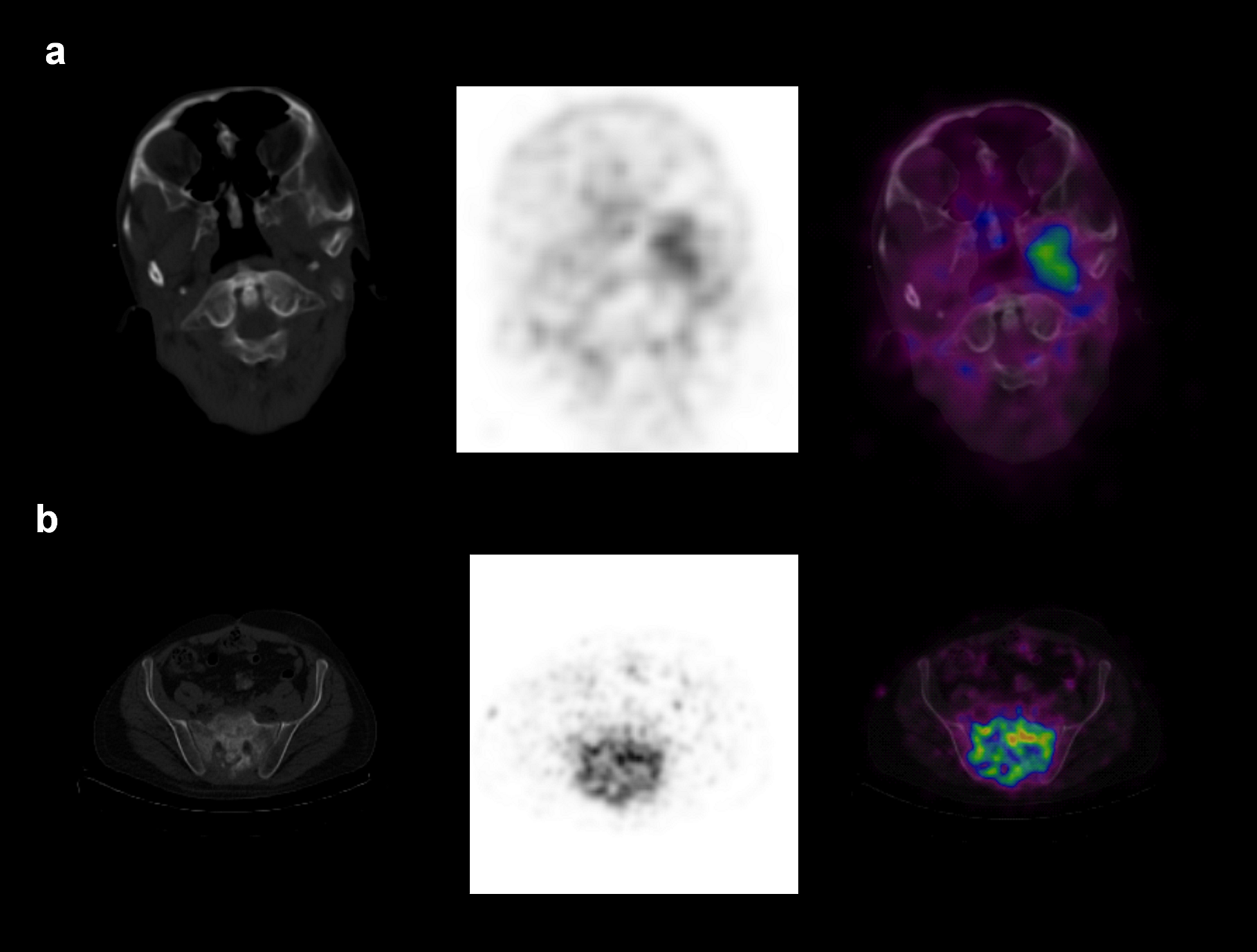

Supplement: Supplementary file 5 — Additional examples of focal tumour uptake of 89Zr-RG7356 at 96 h p.i.. From left to right: low dose CT, attenuation-corrected PET and fused image. (a) Tumour lesion: left side of the skull (patient 8, 450 mg cohort). (b) Tumour lesion: sacrum (patient 13, 675 mg cohort). (TIFF 6335 kb) [file 13550_2018_358_MOESM5_ESM.tif]
